# Observations on the Use of Amalgam

**Published:** 1849-10

**Authors:** H. Colburn


### ARTICLE II.

*Observations on the Use of Amalgam.* By H. Colburn, M. D.

It is an old maxim, that a "drowning man will catch at a straw," and some of the advocates of amalgam give the best possible illustration of its truth. That there are well meaning, honest men who advocate its use, I doubt not, and such may be both Mr. F. H. Clark and Mr. G. Jones, who figured in the September number of the New York Dental Recorder; but it will be necessary for them and other advocates of amalgam, to produce better reasons than their communications contain, to induce men of sound minds, properly educated for their professions, to stop up a decayed tooth with mercury under any circumstances, more particularly where it is so much diseased that the patient cannot bear the removal of the decay. That Mr. Jones had a perfect right to have his tooth filled with it, or any other material, I presume no one will pretend to deny, (unless the question of a right to attempt suicide be raised,) but when such a theory as his letter contains is published to the world as worthy of imitation by experimentalists, (and in the dental profession they are legion,) every reflecting man must see at a glance what disastrous consequences will almost necessarily be the result. If Mr. Jones' theory of knowingly shutting up decay in a tooth, proceeds from his "knowledge of *teeth*, gained from a study of the medical sciences," he has probably also learned, that gangrene, or a scrofulous ulcer may be checked, perhaps cured, by covering over the diseased part with a strip of adhesive plaster. Who can doubt for a moment that his name is destined to occupy an illuminated page in the records of medical science?

By publishing the letter of Mr. Jones, Mr. Clark has assumed no small responsibility. Place amalgam in the hands of any person, child or adult, and he could do all that was done for Mr. Jones, aye, Mr. J. might as well have performed for himself. Such being the case, all that would be wanting to make "every man his own dentist," would be to teach him where to find *that ligament*, put a pair of forceps in his hands, a "little silver and quicksilver," and the parent is prepared to operate upon the child, and *vice versa.* All will then be "regulars," and Mr. Jones will have the gratification of knowing that he is the great regulator.

Scarely a day passes that the intelligent dentist is not an unwilling witness of false theories. It is a misfortune that our profession labors under, that they can be practiced upon without the ability, on the part of the patient to detect them, until he finds that he has been victimized.

One would suppose that the numerous unpleasant results on record, from the use of amalgam, would be sufficient to induce every person to discard it from his practice; but each appears to think *his* amalgam, and *his* mode of using it, is perfectly safe, and that it is a great blessing to mankind that they should enjoy the benefit of *his* services; but I am apprehensive that not a few of them are like Dr. Sangrado.

One of this class of gentlemen, whose intentions I am sure are strictly honest, took considerable pains to impress upon my mind that *his* preparation was wholly innoxious, and referred me to cases that had proved highly satisfactory to his patients, and to one lady in particular, whose *front teeth* had all been saved by it, they being so much decayed that they would not bear a gold filling. Three of his former patients have placed themselves under my care.

The first was a boy of fifteen years of age, and the first glimpse I had of the first inferior right molar induced me to think that about one-third of it consisted of decay; but on examination I found it was filled with amalgam, (the pure "royal" suck-'em-in,) and surrounded by decay. The filling was removed with difficulty, on account of tenderness it had occasioned, and the removal of the decay proved very painful, although the pulp-cavity was not exposed. The refilling, which was done with gold, was almost unbearable, owing to the great sensitiveness of the organ, and its finishing was delayed for a few days from the same cause.

The filling has been in for nearly two years, with a fair prospect that the tooth is saved to the patient; but for some months the tenderness existing in it made it an exceedingly doubtful case.

The second case was that of a gentleman between thirty and forty years of age, for whom I filled a number of teeth, and who had had the "royal" inserted in four or five others, about as many years ago, each of which presented a blackened surface. The lateral surface of the first superior right bicuspid had been filled with it, and the anterior surface was decayed. This tooth had given my patient some trouble, from time to time, but not much. I hesitated what to do. I was privileged to remove the amalgam if I thought it advisable to do so. I stated that I should certainly do so if it were in my own family; but I disliked so much the appearance of wishing to make a case, that I would put in the filling without its removal. I went on with the excavation until I found that I had a clean white cavity, and well shaped. I then took my magnifying glass, (as I am in the habit of doing to examine all cavities where it can be used,) and I was somewhat surprised at finding some small shining globules at its base. I wiped out the cavity again with cotton, but they were still there. I resorted to my excavator again and again, but they were still visible, and I became satisfied that they were "a little quicksilver" imbedded in the substance of the bone immediately over the pulp-cavity. The tooth had now become quite sensitive, and I decided to postpone the filling, having no desire to galvanize my patient. I used a solution of chloroform and gum copal for a temporary filling, and three days afterwards removed it, when the globules being no longer visible, and the tenderness having subsided, I filled the cavity with gold. This was done August 17th and the tooth gave no trouble to the patient for more than two weeks, when it became very sore and painful. After bearing it for four or five days, he called on me and stated the fact. In the mean time my friend's third patient had come under my care with a similar tooth, and I advised the second to bear with his for a few days, until the third was disposed of, who was then under treatment.

The third patient was a young man, nineteen years of age. My amalgam friend had filled the second superior right bicuspid with *his* paste, and the corresponding tooth on the other side with gold. Here I had an opportunity of witnessing the superiority of amalgam fillings over gold, in the hands of *some* persons. The surface of the former was not oxydized, although it had darkened the tooth in its vicinity; but the gold filling presented a concave surface, the edges being below the enamel, and what remained of it was very soft. The secret of his attachment to amalgam was readily solved. He could stop up a cavity with the mercurial paste, but did not understand how to fill one properly with gold, and he was readily induced to prefer the former. Both of the fillings were on the anterior surfaces, near the gum. The one filled with amalgam had been sensitive for some months, and required filling in the lateral surface. I removed the decay, but so sensitive was the tooth that a second sitting was necessary to prepare the cavity for a filling. I did not detect, as in the previous case, small globules of a "little quicksilver," but found I was obliged to delay the filling for some days after the cavity was prepared, for the tenderness to subside. I made use of the same temporary filling as before. The tenderness having in a great measure ceased, I filled the tooth. It still gave my patient trouble, and I resolved on the removal of the "royal," which I did, and found that it had not preserved the organ from decay. Having removed the latter, I put in a gold filling, and am happy in knowing that the sensitiveness, which had existed for months, decreased daily, until food could be masticated without difficulty, and that my patient is now entirely relieved.

Now, Messrs. Editors, what do these facts prove?

Do they not plainly show the deleterious effects of amalgam? Does not every medical man know that there is no agent more destructive to the teeth than mercury? And yet, one who professes to have some "knowledge of the teeth, gained from a study of the medical sciences," would have us believe that it may be used with impunity for fillings. I am well aware that many persons may be found who have had their teeth filled with amalgam, who will assert, (and perhaps some of them truly,) that they have never realized any inconvenience from it; but the probability is, that the greater number of them, if carefully questioned, would give testimony that would be sufficient to condemn its use.

Constitutions are so very differently affected by mercury, some persons being salivated by any quantity given them, large or small, while others take it frequently for years without any apparent injurious result, that it is necessary to be cautious at all times in its use, otherwise, immense mischief may be produced.

I will briefly state a case in point. A girl about twelve years of age, the daughter of a friend, had a bilious attack, about a year ago, and a physician being called in, four or five grains of calomel were administered. Contrary to the wish of the medical attendant, ptyalism was produced. Being at the time a short distance in the country, she was brought home to the city, and placed under the care of two respectable physicians. In a few days gangrene supervened; and where do you suppose it first manifested itself? She had a decayed molar tooth, and *in* and around *it* was mortification first developed; rapidly extending upwards towards the eye, destroying both the osseous and soft tissues in its course. The eye lost, it attacked the nose, and finally, the remaining eye, which was on the point of falling out, when death came to the poor sufferer's relief.

How far will this case suit the advocates of amalgam? Is it necessary that numerous other painful cases should be presented to them, before they can see that the teeth and their appendages are so very susceptible to the influences of mercury, that they can never be sure what effect it may produce on a patient?

I do not condemn the use of mercury in the hands of a judicious physician. On the contrary, I consider it one of the most valuable of all therapeutical agents; but its abuse is what I would protest against, and it is because I believe it always a dangerous experiment to fill a tooth with it, (it certainly is never necessary,) that I have noticed the letters in the Recorder. Having no personal knowledge of either of the gentlemen named, I have no cause for unkind feeling toward them. It is against the system, rather than individuals, that I have aimed.

Should you, Messrs. Editors, think these reflections of sufficient interest to occupy a page in the Journal, they are at your service.

BALTIMORE, *September* 20, 1849.

